# MINDFULNESS TRAINING FOR CAREGIVERS OF ALZHEIMER PATIENTS: PRELIMINARY RESULTS OF A RANDOMIZED CONTROLLED TRIAL

**DOI:** 10.1093/geroni/igz038.3159

**Published:** 2019-11-08

**Authors:** Miriam Hurtado Pomares, Daniel Mendialdua Canales, Paula Peral Gómez, Cristina Espinosa Sempere, Iris Juárez Leal, Desireé Valera Gran, Eva Mª Navarrete Muñoz, Alicia Sánchez Pérez

**Affiliations:** 1 UNIVERSIDAD MIGUEL HERNÁNDEZ DE ELCHE, Elche (Alicante), Spain; 2 Miembro de la Orden del Interser, San Juan de Alicante (Alicante), Spain; 3 UNIVERSIDAD MIGUEL HERNÁNDEZ DE ELCHE, San Juan de Alicante (Alicante), Spain

## Abstract

Mindfulness Based Health Care (MBHC) provides meditation techniques that may help to mitigate negative impact of dementia caregiving. We conducted a pilot randomized controlled trial with two parallel groups including 42 caregivers of Alzheimer patients (21 pairs). MBHC group learned the practice of mindfulness meditation once a week through 8 classes; control group did not receive any therapy. Anxiety and depression symptoms were the primary outcomes assessed in two occasions, i.e. at baseline and the end of the program, using the Hospital Anxiety and Depression scale respectively. Neuropsychiatric disorders of Alzheimer patients were also measured with Neuropsychiatric Inventory-Questionnaire. The details of the randomized controlled trial were described in clinicaltrial.gov NCT03858283. In this abstract, we presented the results of a pilot study conducted with 42 caregivers. Robust linear regression using MM-type estimator was performed to evaluate the changes in neuropsychiatric disorders in patients and anxiety and depression symptoms in caregivers adjusting for Score of Scale Global Deterioration. We observed that, compared to the control group, the MBHC group showed a tendency to a reduction in the total score Neuropsychiatric Inventor (β=-5.20; CI 95%: -10.47; 0.07) and the depression symptoms in the caregivers (β=-2.30; CI 95%: -5.43; 0.83). However, no changes were observed in the anxiety symptoms of the caregivers. In conclusion, the results of this pilot study suggested a positive effective on the reduction of neuropsychiatric symptoms in patents as well as on the depression in the caregivers. Nevertheless, these findings should be confirmed in a further complete study.

